# IgE serum concentration against airborne fungi in children with respiratory allergies

**DOI:** 10.1186/s13223-016-0128-y

**Published:** 2016-04-27

**Authors:** Geusa Felipa de Barros Bezerra, Denise Maria Costa Haidar, Marcos Antonio Custódio Neto da Silva, Walbert Edson Muniz Filho, Ramon Moura dos Santos, Ivone Garros Rosa, Graça Maria de Castro Viana, Luís Zaror, Maria do Desterro Soares Brandão Nascimento

**Affiliations:** Postgraduate building of the Center for Life Sciences and Health, Federal University of Maranhão, Portuguese Avenue, 1966, Bacanga, São Luís, MA CEP 65080-805 Brazil; Maternal and Child University Hospital, Street Barão of Itapary, 227, Center, São Luís, MA Brazil; Universidade Mayor de Temuco, Valdivia, Chile

**Keywords:** Respiratory allergy, Airborne fungi, IgE

## Abstract

**Background:**

To evaluate total and specific E immunoglobulin (IgE) antibody concentrations in underage subjects with respiratory allergic diseases.

**Methods:**

This study was a transversal-type study in 100 underage subjects between 4 and 14 years old, with asthma and/or allergic rhinitis. Total and specific IgE were quantified for airborne fungi in the city of São Luís, Maranhão, Brazil. Five distinct regions—North, South, Center, East and West—were selected so fungi could be collected monthly for 1 year. Twenty genera were identified. *Aspergillus, Penicillium, Fusarium and Neurospora* were selected for the preparation of sensitizing antigens from ELISA dishes. IgE total concentrations were estimated using the same method.

**Results:**

IgE total serum concentration was increased in 97 % of the atopic subjects: 75 % of the subjects presented increased IgE anti-*Aspergillus* concentrations, 87 % presented IgE anti-*Penicillium*, 45 % presented IgE anti-*Fusarium,* and 46 % presented IgE anti-*Neurospora.*

**Conclusions:**

Atopic subjects presented simultaneous IgE total and specific elevations for the tested fungi, possibly due to polysensitization caused by the presence of fungi in all of the areas all year. However, determining the clinical significance of the results was not yet possible because most of the data were isolated variables.

## Background

Respiratory disorders are higher prevalent in children. The different manifestations of allergic diseases involve the upper and lower respiratory airways.

Fungi are important pathogens associated with airway diseases. They interact with epithelial cells, induce increases in inflammatory cytokines, which enhance IgE production, and increase toll-like receptor (TLR) expression [1, 2]. Exposure to fungi can have adverse effects on human health through serious immune responses, infections of several organs and the irritating toxic effects of their subproducts [3].

Fungi and fungal-like organisms are the cause of a lot of diseases in humans and animals, but are also economically important [4]. *Alternaria, Aspergillus, Cladosporium, Fusarium, Penicillium, Stachybotrys* are producers of mycotoxins and allergens that cause respiratory diseases [5].

Allergic rhinitis and allergic asthma are characterized as type 1 allergy. Type 1 allergy is caused by a large number of fungi [6]. Allergic rhinitis is characterized by frequent episodes of sneezing, rhinorrhea, pruritus and nasal obstructions. It is induced by a large number of fungal species, with *Alternaria, Aspergillus, Bipolaris, Cladosporium, Curvularia* and *Penicillium* being the most prominent [6].

Allergic asthma is a respiratory disease characterized by exposure to environmental agents, which provoke allergic inflammation and transitory bronchiolar obstruction, resulting in typical symptoms of coughing and dyspnea [7]. In children, studies conducted by Halonen et al. [8], Nelson et al. [9] and Peat et al. [10] demonstrated that fungal allergy was shown to be associated with increased bronchial reactivity. The genera *Alternaria, Aspergillus, Cladosporium, Helminthosporium, Epicoccum, Aureobasidium* and *Penicillium* have frequently been implicated in allergic asthma [11–13].

Before the outcomes of experimental models, asthma was considered to be a disease caused by IgE because it only occurred due to antigen–antibody complexes formed in mastocyte membranes [12]. Most recent studies have shown that T cells actively participate in asthma manifestation and can cause airway obstruction and allergic inflammation due to the release of cytokines, such as IL-4 and IL-13, which are associated with mastocyte and eosinophil activation [13, 14]. Elevated IgE is commonly found not only in allergic manifestations but also in intestinal and cutaneous parasitosis, acquired or congenital immunodeficiency, viral infections and neoplasias [15, 16]. Also, asthma risk is influenced by genetics. Having a parent with asthma doubles a child’s risk of asthma, and having 2 affected parents increases the risk fourfold. [17].

Indoor dampness increases the risk of indoor fungal growth. The indoor fungal concentrations and species diversity could improve the risk of having asthma, exacerbation of asthma symptoms, or both. *Penicillium* and *Aspergillus* species propagules have been shown to increase the risk of asthma development in children. [18, 19].

In general, outdoor fungi are generally more abundant than indoor fungi, but the association between outdoor fungi and child asthma exacerbations has not been clearly established [20]. The fungal species *Alternaria, Aspergillus, Aureobasidium, Cladosporium, Epicoccum, Helminthosporium* and *Penicillium*, are most frequently implicated in fungi-related allergic asthma exacerbations amongst adults in indoor and outdoor settings [6].

In Brazil, Mezarri et al. [21] found a higher prevalence of the fungi *Cladosporium* (17.86 %) and *Aspergillus/Penicillium* (15.03 %) in the atmospheric air of Porto Alegre, similar to other countries, such as France, Chile, USA and Cuba. Mendes et al. [22] mainly identified *Aspergillus, Botryodiplodia* and *Curvularia* in Aracaju.

The current research aimed to isolate and extract the most common airborne antigens to detect specific IgE concentrations in the serum of atopic subjects, and the expectation was that these concentrations would be correlated according to the patients’ sex, age and place of residence. By doing so, the findings could actually contribute to better control of the disease.

## Methods

### Area of research

The island of São Luís, which is the capital of Maranhão State, Brazil, is in the center of the Maranhão seashore, at 2°20′00′′S to 2°45′00′′S and longitude 44°01′21′′W to 44°24′54′′W. It is located in the north of the state, bordering the Atlantic Ocean; it stretches south to Estreito dos Mosquitos, which separates it from the continent, east to São José Bay and west to São Marcos Bay. It features hot and humid weather and a steady temperature around 27–33 °C [23].

### Sample calculation

To calculate the sample size, Epi Info 3.4.2 (2007, Atlanta, GA) statistical software was used. The calculation was based on the prevalence of asthma in the reference service at 5 % significance.

### Patients

The study sample consisted of 100 patients (64 % boys and 36 % girls), between 4 and 14 years old enrolled in the Pediatric Pneumology Program, University Hospital, Federal University of Maranhão (HUUFMA). Through-chip protocol, clinical data were obtained from patients in the period from 2007 to 2008.

From these subjects, 10 ml of total blood was collected by intravenous puncture after the parent’s/tutors signed the instrument of free consent. The serum was separated and aliquoted for storage in a freezer at 20 °C until the moment of performing the techniques.

### Isolation and airborne fungi identification

Airborne fungi were monthly collected by Bezerra [24], in five areas (North, South, Center, East and West) in the city of São Luís, Maranhão, Brazil, from January to December 2007.

Fungal spores were collected by passive gravitational settling on Petri dishes containing Sabouraud agar, 1.5 m height, for 15 min [21, 25]. A total of 1510 colony forming units (CFUs) were recovered, corresponding to 20 fungi genera. The species will be identified later by microculture technique and electronic microscopy.

Only the genera *Aspergillus, Penicillium, Fusarium* and *Neurospora* showed detectable amounts of protein and were used as antigens to sensitize ELISA plates to investigate the concentration of specific IgE by ELISA index (EI) [26].

### Antigenic extraction

The protocol for the extraction of antigens from filamentous fungi was performed as follows, based on the literature [27, 28] with certain modifications: the fungi were cultivated in an appropriate medium (Sabouraud ágar) for 5 days of culture before washing five times with phosphate-buffered saline (PBS) and transferring the culture to screw-cap tubes containing previously sterilized glass beads and PBS and fungus at a ratio of 3:1. The tubes were vortexed for 15 min and centrifuged for 1 h at 14,500 rpm in a refrigerated centrifuge The supernatant was collected, and the protein was quantitated. The stock of crude extract was aliquoted and stored at 20 °C until use. Stocks were diluted to 50 µl/ml in PBS before use.

The sera from 100 patients with clinically characterized respiratory diseases were subjected to ELISA to detect the concentrations of total and specific IgE against fungi.

Polystyrene plates with 96 wells containing immobilized monoclonal antibodies of the anti-IgE type formed specific complexes with serum IgE through high-affinity bonds. The plates were then washed to remove proteins that had not interacted. A secondary antibody conjugated to peroxidase was added to form a ternary complex with the immobilized.

IgE, followed by washing. Next, 3-30,5-50-tetramethylbenzidine and hydrogen peroxide were added to the solution, which under the action of the immobilized peroxidase, produced a blue color. A strong acid was added to the mixture to halt the reaction, producing a yellow color. A reading was performed at a wavelength of 450 nm. The absorbed intensity was proportional to the concentration of IgE in the sample.

Optical densities were determined spectrophotometrically at 450 nm. The rates of antibodies were expressed as the EI, according to Alves et al. [29]. The EI is calculated by the ratio between the mean optical density of the study group and the optical density of three samples of sera from subjects with no specific IgE (multiplied by 3d, where d is the average standard deviation of optical densities of sera from the group of non-allergic individuals).

EI values equal to or higher than 0.102 (EI ≥ 0.102) were considered to be positive for total IgE; 0.603 (EI ≥ 0.603) for IgE anti-*Aspergillus* spp.; 0.466 (EI ≥ 0.466) for IgE anti-*Penicillium* spp.; 0.298 (EI ≥ 0.298) for IgE anti-*Neurospora* spp.; and 0.565 (EI ≥ 0.565) for IgE anti-*Fusarium* spp.

The cut-off was calculated by averaging the increased negative samples three times to the standard deviation of these samples, where the number of standard deviations used in the formula ensures the confidence level of the result. Cut-off = μ DO + 3 × s [30].

### Ethical aspects

This research was approved by the ethics committee of University Hospital under number 406/06. The Informed Consent Term (ICF) was signed by the parents or guardians of minors who participated in this research.

### Statistical analysis

Statistical analysis was performed using the software Stata/SE 9.0 for Windows (Stata Corporation, College Station, TX). The study was cross-sectional and observational, and descriptive statistical techniques were used to assess all of the variables, with the aid of graphs and tables of frequencies. The Pearson correlation was used to study the relationship between total IgE levels and age (years). The correlation coefficient Phi was used to verify the correlation between positivity for two fungi concomitantly. We considered a p value <0.05 as the significance criterion.

## Results

We studied 100 patients (67 % boys and 33 % girls), between 4 and 14 years old enrolled in the Pediatric Pneumology Program, University Hospital, Federal University of Maranhão (HUUFMA).

A total of 100 patients with a diagnosis of asthma were studied. In total, 45 (45.0 %) of these patients had rhinitis, and 35 (35.0 %) had sinusitis.

Table 1 shows the demographic data. Of the 100 patients studied, 67 (67.0 %) were male and 33 (33.0 %) were female. The mean age was 8.6 ± 2.6 years. In total, 100 % were from an urban area. In all, 19 patients (19.0 %) lived in the North Zone of São Luis; 6 (6.0 %) lived in the South Zone; 35 (35.0 %) lived in the East Zone; 17 (17.0 %) lived in the West Zone and 23 (23.0 %) lived in downtown area. Of the 59 children aged 4–9 years, 20 are female and 39 male, and of the 41 children in the age group 10–14 years, 13 are female and 28 male.

**Table 1:** Demographic and clinical characteristics of the sample of cases

Variables	Cases (n = 100)
Age, years	
Genre	8.6 (± 2.6)
Female	33 (33.0)
Male	67 (67.0)
Residence site	
Urban area	100 (100.0)
Rural area	0 (0.00)
Region	
North	19 (19.0)
South	6 (6.0)
East	35 (35.0)
West	17 (17.0)
Central	23 (23.0)
Sensitization to fungi	
*Neurospora*	46 (46.0)
*Fusarium*	45 (45.0)
*Aspergillus*	75 (75.0)
*Penicillium*	87 (87.0)
IgE total	1.05 (± 0.8)
rhinitis	45 (45.0)
sinusitis	35 (35.0)

Observing Table 2, it is possible to observe that isolated fungus occurrences varied slightly over the year, considering the great rain variation between the rainy season (January–June) of 1702.25 mm^3^ and the dry season (July–December) of 35.50 mm^3^. The median number of CFUs/dish in the rainy season (January–June) was 20 (maximum = 279 and minimum = 0), and the corresponding figure for the dry season was 14 (maximum = 227 and minimum = 0) using the Mann–Whitney test p = 0.96.

**Table 2:** CFU fungus distributions during the year, compared to rain index and the areas of collection in São Luís, MA, Brazil

Months	Rain index (mm^3^)	Isolated colonies/region					Colonies total (CFU)
		N	S	E	W	C	
January	28.25	15	10	20	19	38	102
February	381	15	10	18	19	15	77
March	274.5	12	14	63	27	13	129
April	568.25	17	30	22	19	25	113
May	228	29	12	22	62	44	169
June	97.75	18	19	44	28	9	118
July	114.5	12	25	13	40	22	112
August	0.25	47	43	41	25	32	188
September	0	15	53	21	21	7	117
October	0	25	86	19	28	9	167
November	0	37	17	14	9	8	85
December	35.25	14	38	29	41	11	133
Total	1727.75	256	357	326	338	233	1510

*N* north, *S* south, *E* east, *W* west, *C* center

Twenty genera of fungi were isolated in this study. The main genera found in all of the regions were *Aspergillus* (33.5 %), *Penicillium* (18.8 %), *Cladosporium* (14.2 %), *Curvularia* (10.6 %) and *Fusarium* (7.6 %). A comparison of the average number of CFUs for the five most frequent fungi using Tukey’s test, showed that the number for *Aspergillus* differed significantly (p < 0.05) from the numbers for the genera *Cladosporium, Curvularia* and *Fusarium.*

*Aspergillus* and *Penicillium* were frequently isolated in all of the areas studied, regardless of the month of the year. It was possible to see that, in the Central area, the lowest CFU number (115) was noted; in contrast, in the East area, the highest figures were measured (228) (Table 3).

**Table 3:** Airborne fungi according to regions collected in São Luís, MA, Brazil

Genus	Region					Total
	N	S	E	W	C	CFUs
*Neurospora*	1	5	8	7	0	21
*Fusarium*	11	50	16	30	8	115
*Aspergillus*	96	105	118	109	78	506
*Penicillium*	73	45	86	51	29	284
Total	181	205	228	197	115	926

*N* north, *S* south, *E* east, *W* west, *C* center

In this study, we used the genders *Aspergillus, Penicillium, Fusarium* and *Neurospora* as antigens.

### Detection of total and specific IgE for fungi antigens

**Fig. 1:**
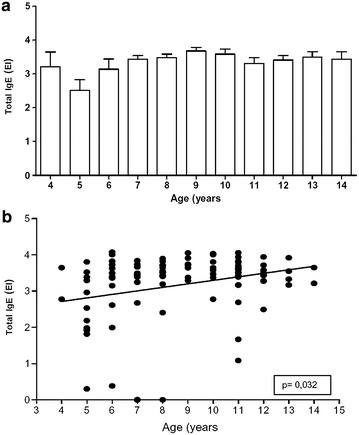
IgE distribution values according to the age (1st) and correlation between their ages and IgE total concentration (*EI* ELISA Index), in São Luís-MA, Brazil, 2007 (p < 0.05). Results in **a** mean ± standard deviation and in **b** individual data in 100 children

Specific IgE antibodies against fungi (anti-*Neurospora*, anti-*Fusarium*, anti-*Aspergillus* and anti-*Penicillium* IgE) were screened in the afore mentioned 100 blood samples. Among the 100 patients enrolled in the Pediatric Pneumology Program, 97 (97 %) demonstrated that IgE total concentrations were detected (Fig. 1a): 64 % boys and 33 % girls. There was a statistically significant difference in IgE total concentration and age (p < 0.05) (Fig. 1b), but not between IgE total concentration and area of residence (North, South, East, West and Center) (p = 0.88).

Among the 100 patients enrolled in the Pediatric Pneumology Program, 75 % were sensitized to *Aspergillus,* 87 % were sensitized to *Penicillium*; 46 % were sensitized to *Neurospora* and 45 % were sensitized to *Fusarium*.

Of the 59 children aged 4–9 years, 93.2 % tested positive for total IgE, 78 % tested positive for IgE anti-*Neurospora*, 76.2 % tested positive for IgE anti-*Fusarium*, 76.2 % tested positive for IgE anti-*Aspergillus* and 84.7 % tested positive for IgE anti-*Penicillium*. Of the 41 children in the age group 10–14 years, 100 % were positive for total IgE, 68.3 % tested positive for IgE anti-*Neurospora*, 68.3 % tested positive for IgE anti-*Fusarium*, 68.3 % tested positive for IgE anti-*Aspergillus* and 87.8 % tested positive for IgE anti-*Penicillium*.

Sex (p = 0.86), local of residence (p = 0.90) and age (p = 0.19) were not statistically significant with anti-*Aspergillus* IgE antibodies. There wasn’t a statistically significant difference in anti-*Penicillium* IgE antibodies and sex (p = 0.39), age (p = 0.47) and local of residence (p = 0.13).

**Fig. 2:**
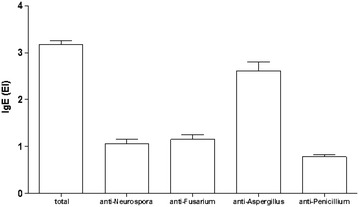
Total IgE, anti-*Neurospora*; anti-*Fusarium*; anti-*Aspergillus* and anti-*Penicillium* concentration IgE, in children´s serum (n = 100) with respiratory allergies treated in a public service in São Luís-MA, Brazil in 2007. Results correspond to mean ± standard deviation

Figure 2 shows the seropositivity to fungi of Total IgE, anti-*Neurospora*; anti-*Fusarium*; anti-*Aspergillus* and anti-*Penicillium* concentration IgE, in children´s serum. A prevalence of seropositivity to fungi was noted, particularly for the genera *Aspergillus* (Fig. 2).

## Discussion

Fungi are important airborne allergens. *Aspergillus* spp. was the most commonly isolated genus in the current study, and *Penicillium* spp. was the second most commonly isolated genus. *Aspergillus, Penicillium*, *Cladosporium, Curvularia* and *Fusarium* are the most frequent outdoor species, according to previous researches [31, 32]. Research conducted in Mexico city showed that *Penicillium* spp. is also the second most common genus [33]. In Brazil, nostheast cities, like Fortaleza, Natal and Recife are climatically similar with São Luís. In Recife and Natal, *Aspergillus* and *Penicillium* were more frequente genera [34, 35]. In Fortaleza, *Curvularia* spp. was the most isolated fungi [25].

IgE antibody concentrations in serum vary according to age and as a consequence of contact with antigens, which is also common among the other immunoglobulin classes. In this research, these concentrations were higher in boys regardless of age. Spalding et al. [36] demonstrate that the concentration of IgE is higher for males in all age groups. Our results corroborate these authors, since the increase of IgE occurred in 64 % of males and 33 % females.

Specific IgE dosing is one of the most important in vitro methods for the diagnosis of immediate hypersensitivity [37]. In the current research, increased IgE concentrations were observed in 96.9 % of the patients. These figures were higher than those described by Caballero et al. [38], who evaluated 35 patients aged between 3 and 16 years old aged with respiratory allergies and noted elevated total IgE in 77.2 % of the sample.

Atopic patients generally have specific IgE antibodies for several antigens, including fungi, as part of polysensitization. Allergic reactions and inhaled antigens have frequently been reported, although there has not been any proof that exposure to fungi transported by the air in outdoor environments plays any role in allergic rhinitis or other allergies of superior airways [3].

Among the 100 patients enrolled in the Pediatric Pneumology Program, 97 (97 %) demonstrated that IgE total concentrations were detected. 75 % were sensitized to *Aspergillus,* 87 % were sensitized to *Penicillium*; 46 % were sensitized to *Neurospora* and 45 % were sensitized to *Fusarium*.

A Mexican study involving pediatric patients with respiratory allergy showed that the most frequent allergens were *Rhizopus, Aspergillus, Cladosporium* and *Candida*. Total IgE was found to be high in 77.2 % of patients, and specific IgE was found to be high in 31.4 % [38].

In Malaysia, Goh et al. [39] found that 72.73 % of subjects had positive specific IgE against *Penicillium*. The result is similar to that of the patients in our study (87 %), can be explained by the use of ELISA in both studies and the high frequency of *Penicillium* in Brazil and Malaysia. According to Burge et al. [40], *Penicillium* is common in outdoor and indoor environments. Patients with asthma usually present positive responses for spores and other isolated *Penicillium* antigens.

Osorio et al. [41] found hypersensitivity to fungi, mainly in the genera *Penicillium* and *Aspergillus*, in 12 of 13 asthmatic schoolchildren in their study in the city of Recife. In a study conducted in Taiwan, *Candida* (72.1 %) was the most common sensitizing agent, followed by *Aspergillus* (51.2 %) and *Penicillium (*41.9 %) [42].

There was not statistic correlation with sex and local of residence and IgE total antibody concentrations or specific IgE to *Aspergillus* and *Penicillium* in this study. Considering that *Aspergillus* was the most frequently isolated antigen in this research, and it was also present in all areas of the city all year, it is reasonable to suppose that these fungus spores are naturally sensitizing. However, most of the subjects of this research lived in the central area of the city, which was where the fewest fungi were detected, possibly due to a larger concentration of buildings and homes.

Regarding age, there was statistical significant correlation between age and total IgE, but not between age and IgE anti-*Aspergillus* and anti-*Penicillium.* Chang et al. [42] found that the sensitization pattern revealed that fungal allergy was extremely common among children with respiratory allergic disease, especially in those <10 years of age. One possible reason is that younger children may be less efficient in clearing airway fungal spores, which results in higher and longer antigenic stimulation.

There was no correlation between total IgE and anti-*Aspergillus* IgE in the current research; however, this finding led us to suppose that elevated anti-*Aspergillus* IgE concentrations might have contributed to the elevation of serum total IgE.

### Strenghts and limitations

This study with a large number of patients sensitized to *Aspergillus and Penicillium,* or less frequently to *Fusarium* and *Neurospor*a spp., shows the importance of these sensitisations.

However, this study has some limitations. First, we have not accounted or discounted the possibility that the findings could be a result of indoor exposures on elevated IgE. In addition, the sample included only 100 children. The technique used to assess the levels of IgE ELISA was the index and not by Phamacia CAP unit system. Limitations in the statistical analysis. This is a cross-sectional study and there was no assessment of the levels of exposure to fungi and the development of allergic diseases. There was not a control group. Besides, the prick test was not conducted to confirm allergy to fungi of the study.

## Conclusions

The genera of fungi identified in the present study were correlated with natural systems and could be useful in assessing the impact of environmental changes on the region studied.

In short, based on the data obtained in this study, the following aspects of the causal relationship between exposure and the symptoms of respiratory allergy may be noted. This study shows the importance of airborne fungi sensitizations in children with respiratory allergy. Combined diagnosis of total IgE test, specific IgE and clinical aspects is very useful for deciding specific therapies.

Finally, it was possible to conclude that the presence of specific IgE antibodies for the studied airborne fungi might be due to polysensitization because the fungi were present in all of the areas over the whole year in the city of São Luis-MA, Brazil, so the topic requires more study to understand fully airborne fungus respiratory allergies.

## Abbreviations

IgE: E immunoglobulin
LIF: immunophysiology laboratory
NIBA: nucleum of basic and applied immunology
